# Supplementary material for: Effect of individual variations in genes related to dopamine brain transmission on performance with and without rewards during motor sequence and probabilistic learning tasks in children and young adults with and without cerebral palsy
Source: PLoS One. 2025 Jan 9;20(1):e0314173. doi: 10.1371/journal.pone.0314173 (PMC11717210; doi:10.1371/journal.pone.0314173)
Supplement: S1 Table — (DOCX) [file pone.0314173.s001.docx]

Supplemental Table 1. **Calculation of Individual Gene Scores.**

| **Polymorphisms with associated gene scores** |
| --- |
| DRD1 rs4532 DRD2 rs1800497 DRD3 rs6280 COMT rs4680 DAT rs28363170  Glu/Glu = 1 A/A = 0 Ser/Ser = 0 Val/Val = 0 9/9 = 1  Glu/Lhys = 0 A/G = 1 Ser/Gly = 1 Val/Met = 1 9/10 = 1  Lys/Lys = 0 G/G = 1 Gly/Gly = 1 Met/Met = 1 10/10 = 0 |

The presence of polymorphisms in dopamine-related genes determined for each participant and then summed to yield a total score for each individual (15).
