# Supplementary material for: Effect of individual variations in genes related to dopamine brain transmission on performance with and without rewards during motor sequence and probabilistic learning tasks in children and young adults with and without cerebral palsy
Source: PLoS One. 2025 Jan 9;20(1):e0314173. doi: 10.1371/journal.pone.0314173 (PMC11717210; doi:10.1371/journal.pone.0314173)
Supplement: S2 Table — (DOCX) [file pone.0314173.s002.docx]

Supplemental Table 2. **Effect of individual genes.**

|  | BDNF | COMT | DRD1 | DRD2 | DRD3 | DAT |
| --- | --- | --- | --- | --- | --- | --- |
| SRTT Reaction Time  Condition  Cond. X Gene Group  Gene Group  SRTT Error Rate  Condition  Cond. X Gene Group  Gene Group  WPT Correct  Condition  Cond. X Gene Group  Gene Group  WPT Reaction Time  Condition  Cond. X Gene Group  Gene Group  N for (0/1) Frequency | 0.47  0.98  0.79  0.64  0.60  0.37  **0.09**  0.63  0.63  0.18  0.47  0.47  (5/20) | 0.99  0.24  0.77  0.65  0.68  0.24  0.36  0.36  0.36  0.11  0.84  0.90  (5/20) | 0.87  0.47  0.85  0.26  **0.065**  **0.058**    **0.01***  **0.076**  **0.076**  **0.04***  0.46  0.80  (4/21) | 0.39  0.35  0.34  0.77  **0.07**  0.59  **0.082**  0.95  0.95  **0.035***  0.40  0.40  (13/12) | 0.49  0.56  0.15  0.85  0.17  0.73  0.11  0.76  0.76  **0.055**  0.62  0.62  (9/16) | 0.53  0.19  0.53  0.72  0.58  0.66  0.48  0.74  0.74  0.17  0.83  0.20  (23/2) |

P-values for the main and interaction effects for GLM comparing unrewarded and rewarded reaction time and error rate outcomes across each individual gene score.
