# Supplementary figures and images for: Effect of individual variations in genes related to dopamine brain transmission on performance with and without rewards during motor sequence and probabilistic learning tasks in children and young adults with and without cerebral palsy
Source: PLoS One. 2025 Jan 9;20(1):e0314173. doi: 10.1371/journal.pone.0314173 (PMC11717210; doi:10.1371/journal.pone.0314173)

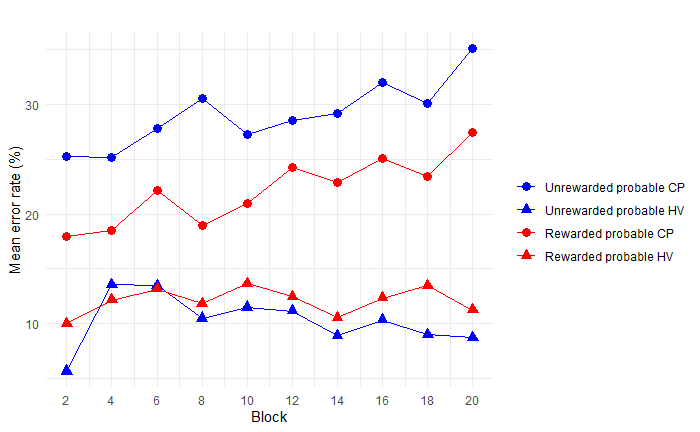

Supplement: S1 Fig — a. Mean change in reaction time per block during training for the group with CP and controls (HV) for the two probable sequences in the unrewarded and rewarded conditions Baseline (unrewarded) scores are relatively worse but show greater changes over time in CP, and there appears to be a baseline shift towards faster reaction times in the rewarded condition. b. Mean change in error raate per block during training for the group with CP and controls (HV) for the two probable sequences for the unrewarded and rewarded conditions Relatively worse baseline scores and increasing errors observed in CP that are lower in the Rewarded condition. The HV group shows a generally flatter pattern and one that shows a slightly negative rather than positive effect from rewards. (ZIP) [file pone.0314173.s003.zip › Supporting information Supplemental fig. 1b.docx]

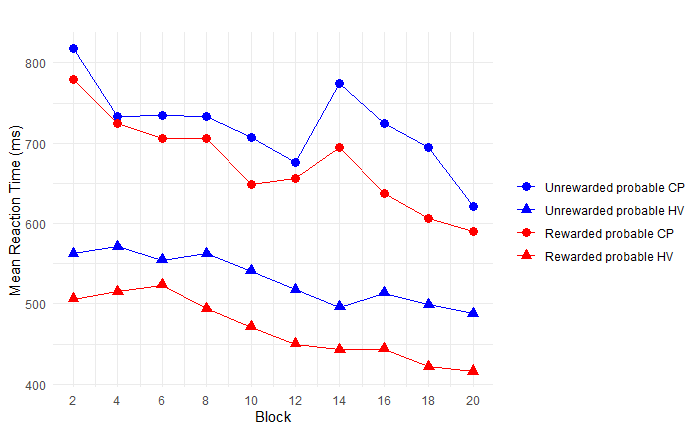

Supplement: S1 Fig — a. Mean change in reaction time per block during training for the group with CP and controls (HV) for the two probable sequences in the unrewarded and rewarded conditions Baseline (unrewarded) scores are relatively worse but show greater changes over time in CP, and there appears to be a baseline shift towards faster reaction times in the rewarded condition. b. Mean change in error raate per block during training for the group with CP and controls (HV) for the two probable sequences for the unrewarded and rewarded conditions Relatively worse baseline scores and increasing errors observed in CP that are lower in the Rewarded condition. The HV group shows a generally flatter pattern and one that shows a slightly negative rather than positive effect from rewards. (ZIP) [file pone.0314173.s003.zip › Supporting information Supplemental fig. 1a.docx]
