# Supplementary material for: Effect of individual variations in genes related to dopamine brain transmission on performance with and without rewards during motor sequence and probabilistic learning tasks in children and young adults with and without cerebral palsy
Source: PLoS One. 2025 Jan 9;20(1):e0314173. doi: 10.1371/journal.pone.0314173 (PMC11717210; doi:10.1371/journal.pone.0314173)
Supplement: Attachment — Submitted filename: Response to Reviewers.docx [file pone.0314173.s004.docx]

**PONE-D-24-30058**

**Effect of individual variations in genes related to dopamine brain transmission on performance with and without rewards during motor sequence and probabilistic learning tasks in children and young adults with and without Cerebral Palsy**

*Thanks to the Editor and to the reviewers for describing what changes were required or recommended and for the overall positive response to the manuscript. We have addressed each comment as detailed below with our responses in italics.*

Dear Dr. Damiano,

Thank you for submitting your manuscript to PLOS ONE. After careful consideration, we feel that it has merit but does not fully meet PLOS ONE’s publication criteria as it currently stands. Therefore, we invite you to submit a revised version of the manuscript that addresses the points raised during the review process.

Journal Requirements:

When submitting your revision, we need you to address these additional requirements.

1. Please ensure that your manuscript meets PLOS ONE's style requirements, including those for file naming. The PLOS ONE style templates can be found at

<https://journals.plos.org/plosone/s/file?id=wjVg/PLOSOne_formatting_sample_main_body.pdf> and 
<https://journals.plos.org/plosone/s/file?id=ba62/PLOSOne_formatting_sample_title_authors_affiliations.pdf>

*We have carefully reviewed all instructions and have made modifications throughout the manuscript to comply with all.*

2. Thank you for stating the following in the Acknowledgments Section of your manuscript:

“This work was funded by the Intramural Research Program at the National Institutes of Health Clinical Center (Protocol # 16-CC-0149). We also acknowledge the help of Dr. Leonora Wilkinson who developed the computer versions of the tasks utilized here.”

We note that you have provided funding information that is not currently declared in your Funding Statement. However, funding information should not appear in the Acknowledgments section or other areas of your manuscript. We will only publish funding information present in the Funding Statement section of the online submission form.

Please remove any funding-related text from the manuscript and let us know how you would like to *update your Funding Statement.*

*The acknowledgement was updated in the text. The funding statement was updated in the Cover letter.*

3. In the online submission form you indicate that your data is not available for proprietary reasons and have provided a contact point for accessing this data. Please note that your current contact point is a co-author on this manuscript. According to our Data Policy, the contact point must not be an author on the manuscript and must be an institutional contact, ideally not an individual. Please revise your data statement to a non-author institutional point of contact, such as a data access or ethics committee, and send this to us via return email. Please also include contact information for the third party organization, and please include the full citation of where the data can be found.

*We have submitted this to the Dryad data repository on 9/18/24 with the following confirmation:*

Relating genetic variations in dopamine brain transmission to task performance with and without rewards submitted with DOI https://doi.org/10.5061/dryad.qnk98sfs5. There may be a delay for processing before the item is available.

4. We notice that your supplementary tables are included in the manuscript file. Please remove them and upload them with the file type 'Supporting Information'. Please ensure that each Supporting Information file has a legend listed in the manuscript after the references list.

*We have created Supporting information files and listed the legends in the manuscript after the references.*

5. Please review your reference list to ensure that it is complete and correct. If you have cited papers that have been retracted, please include the rationale for doing so in the manuscript text, or remove these references and replace them with relevant current references. Any changes to the reference list should be mentioned in the rebuttal letter that accompanies your revised manuscript. If you need to cite a retracted article, indicate the article’s retracted status in the References list and also include a citation and full reference for the retraction notice.

*All are correct – two needed to be added in response to a reviewer’s comment.*

5. Review Comments to the Author

Please use the space provided to explain your answers to the questions above. You may also include additional comments for the author, including concerns about dual publication, research ethics, or publication ethics. (Please upload your review as an attachment if it exceeds 20,000 characters)

Reviewer #1: The manuscript examines the influence of dopamine-related genetic variability on learning in young individuals with and without cerebral palsy (CP). The study calculated individual dopamine-related gene scores and compared these to performance on two tasks: an implicit sequence learning task and a probabilistic classification task. Both tasks were administered in unrewarded and rewarded conditions, the latter known to increase dopamine levels. The key finding was an interaction between gene score and condition for the sequence learning task: individuals with low gene scores performed poorly without rewards but improved with rewards, while those with high gene scores showed the opposite pattern.

The study is exceptionally well-written and demonstrates a comprehensive understanding of the literature. Despite the small sample size, it represents a significant milestone by highlighting the potential for personalized neuro-rehabilitation approaches, showing how dopamine-related genetic differences can influence motor skill learning in young individuals with CP.

I just have a few minor comments:

1. On Page 6, please ensure the correct usage of 'Brain-derived neurotrophic factor (BDNF)

*Thank you for noticing this. We mixed up the variants for COMT and BDNF and have now corrected this.*

2. On Page 20, correct the name 'Diaz Heist' to 'Diaz Heijtz.'

*Done*

3. Please add a footnote to Table I with brief explanations for GMFCS and MACS to help general readers understand these classifications.

*We have now added a brief description to the text with references for each and a footnote in the table to spell out these acronyms.*

Reviewer #2: The authors studied the relationship between dopamine genes, expressed collectively as a gene score, and motor learning using a 2x2 design, consisting of an implicit sequence learning task and a probabilistic classification task, each administered in an unrewarded condition and a rewarded one. Study subjects had CP (n=13) or were healthy controls (n=12). The main finding was an interaction between gene score and condition for the sequence task, such that those with low gene scores were poorer learners without rewards but responded positively to rewards; whereas the converse was true for those with high gene scores. The authors conclude that that genetic variability related to dopamine may influence neurorehabilitation outcomes and could potentially be modulated using rewards or medications for those with poorer learning at baseline, which can promote a more personalized approach to patient care.

The report is well written, and the conclusions are justified by study results.

Were the main significant results significant in CP, healthy controls, or both?

*The main result analyzing gene scores with performance in the rewarded and unrewarded conditions with both groups combined showed a significant interaction between gene score group and SRTT error rates.*

*Other significant results included:*

- *SRTT reaction time improvement was greater for the probable compared to the improbable sequence (as expected)*
- *SRTT error rate increased over time and more for the probable sequence for groups combined*
- *SRTT error rate higher in CP*
- *WPT reaction time slower in CP*
- *WPT reaction time slower in PA compared to FB for groups combined*

*It is not surprising that those with CP tended to move more slowly or make more key press errors, but they had similar learning in response to training as those with HV, and the effect of rewards was not seen in the group as a whole or in either subgroup until gene scores were introduced as a factor.*

The verb tense jumps around between present and future, which could confuse some readers.

*Thanks, we have now carefully checked through the whole document and made changes throughout	the text to be more consistent.*

Subjects were age 5-25, thus: did results vary by subject age?

*We had correlated the results with age and found no relationship with the WPT cognitive task but there was a moderate relationship between age and SRTT reaction time and error rate with younger being slower (r = 0,47; p = 0.02) and making more errors (r = 0.52; p = 0.01) which is not surprising because motor coordination and speed improve with age. We chose not include this for 2 reasons: 1. Long length of the manuscript already and 2. Age did not differ between groups so main results were not impacted.*

*If you think these should be included, we would be happy to do* so.

Was there an order effect with respect to order of reward condition and order of task?

*The order was randomized across subjects to control for this. And as an additional step, when we did a further check and compared results for those who did each first with those who did each second, there was no significant difference.*

Line 305 is “CP vs. TD group”, and the term “TD” is used later. But “TD” is not defined, but it should be.

*Thanks for noticing this. We should have used HV instead as had been used throughout the rest of the manuscript to indicate the control group. “TD” has now been removed and replaced by “HV”.*

Line 367 Notes that “The true influence of gene scores only became apparent when we evaluated the unrewarded and rewarded conditions together.” This indicates that the paragraph with lines 340-351 evaluated the unrewarded and rewarded conditions separately, but this is not clear when reading that paragraph. The authors are asked to clarify these points to ease reader comprehension.

*The paragraph 340-351 had not yet included gene scores as a factor, so the effect of rewards (which was subsequently found to be positive in those with low gene scores and the opposite in those with high gene scores) was washed out when analyzing the group as a whole or CP or HV alone, as was also seen in the Pearson-Fuhrhop results until they looked at the gene scores.*

One limitation that the authors are encouraged to discuss is that the current findings are based on single session learning paradigms. However, neurorehabilitation typically occurs over many sessions, and so some uncertainty exists as to how current findings extrapolate

*This is a good point. As suggested, we noted this difference in the discussion in lines 530-534 but added that our findings were encouragingly similar to those with longer training.*

While revising your submission, please upload your figure files to the Preflight Analysis and Conversion Engine (PACE) digital diagnostic tool, [https://pacev2.apexcovantage.com/](https://gcc02.safelinks.protection.outlook.com/?url=https%3A%2F%2Fc05y1x9s.r.us-east-2.awstrack.me%2FL0%2Fhttps%3A%252F%252Fpacev2.apexcovantage.com%252F%2F1%2F010f0191e3bc4d0d-51584071-ac39-4a99-bcf2-2511f0a8b104-000000%2FQhm0W1i12gOyfHjY_C-KoWYiTgk%3D176&data=05%7C02%7Cdamianod%40cc.nih.gov%7C74510c89f47b45efd2a908dcd2c5eb59%7C14b77578977342d58507251ca2dc2b06%7C0%7C0%7C638616997024508938%7CUnknown%7CTWFpbGZsb3d8eyJWIjoiMC4wLjAwMDAiLCJQIjoiV2luMzIiLCJBTiI6Ik1haWwiLCJXVCI6Mn0%3D%7C0%7C%7C%7C&sdata=V70fAI30PLYri1ikcmUI9w3li%2F1E4tNjLDeppkbf4dc%3D&reserved=0). PACE helps ensure that figures meet PLOS requirements. To use PACE, you must first register as a user. Registration is free. Then, login and navigate to the UPLOAD tab, where you will find detailed instructions on how to use the tool. If you encounter any issues or have any questions when using PACE, please email PLOS at [figures@plos.org](mailto:figures@plos.org). Please note that Supporting Information files do not need this step.

*Done*
