# Supplementary material for: Effect of individual variations in genes related to dopamine brain transmission on performance with and without rewards during motor sequence and probabilistic learning tasks in children and young adults with and without cerebral palsy
Source: PLoS One. 2025 Jan 9;20(1):e0314173. doi: 10.1371/journal.pone.0314173 (PMC11717210; doi:10.1371/journal.pone.0314173)
Supplement: Attachment — Submitted filename: Response to Reviewers.docx [file pone.0314173.s005.docx]

**Response to Reviewers:**

Thanks for your review. We have included the reviewer’s comments and our responses for each below.

Reviewer #2: Two minor requests remain:

Please provide a very brief summary of results regarding group effect (CP vs. healthy controls) and age effect, in the Results section. Each can be quite concise. I understand space constraints, and that complex results can sometimes blur key findings, but future readers of the manuscript will likely also have questions about these two effects.

*We have now added a statement to the results summarizing the single difference found between groups. This had also been stated in the discussion in the previous version of the manuscript. We have also summarized the results of the analysis of age effects to the section where we had previously examined the effects of sex.*

Regarding ordering effect, the authors note “when we did a further check and compared results for those who did each first with those who did each second, there was no significant difference.” Randomization doesn’t always solve things completely, as the authors know, especially with smaller sample sizes, and so reporting this finding will increase the impact of the overall findings. As such, can the authors please add this result to the manuscript?

*We have now included the data analyses for each outcome depending on order, as shown in the table below. We have summarized this in the results along with the results for age and sex, stating that there was no significant effect of order*.  *We have also added a statement on the additional analyses done in the statistical analysis section.*

Table. Mean values for each condition depending on order

| Variable | Rewarded first | Unrewarded first | p value |
| --- | --- | --- | --- |
| SRTT reaction time – U (ms)  SRTT Reaction time – R  SRTT Error rate – U  SRTT Error rate – R  WPT Proportion Correct – U  WPT Proportion Correct – R  WPT Reaction time – U  WPT Reaction time - R | **581.5 ± 269.8**  652.3 ± 294.5  0.25 ± 0.28  0.19 ± 0.20  **0.78 ± 0.13**  **0.72 ± 0.16**  **2793.7 ± 548.1**  2605.5 ± 805.9 | 686.0 ± 242.0  **520.6 ± 125.3**  **0.15 ± 0.15**  **0.15 ± 0.11**  0.69 ± 0.14  0.63 ± 0.18  2820.6 ± 582.1  **2360.0 ± 845.2** | 0.32  0.15  0.29  0.51  0.11  0.18  0.91  0.47 |

U = unrewarded condition; R = rewarded condition

Bold = better score
